# Radiosensitization by the investigational NEDD8-activating enzyme inhibitor MLN4924 (pevonedistat) in hormone-resistant prostate cancer cells

**DOI:** 10.18632/oncotarget.9526

**Published:** 2016-05-20

**Authors:** Xiaofang Wang, Wenjuan Zhang, Zi Yan, Yupei Liang, Lihui Li, Xiaoli Yu, Yan Feng, Shen Fu, Yanmei Zhang, Hu Zhao, Jinha Yu, Lak Shin Jeong, Xiaomao Guo, Lijun Jia

**Affiliations:** ^1^ Cancer Institute, Fudan University Shanghai Cancer Center, Collaborative Innovation Center of Cancer Medicine, Shanghai, 200032, China; ^2^ Department of Radiation Oncology, Fudan University Shanghai Cancer Center, Shanghai, 200032, China; ^3^ Department of Oncology, Shanghai Medical College, Fudan University, Shanghai, 200032, China; ^4^ Department of Immunology, School of Basic Medical Sciences, Fudan University, Shanghai, 200032, China; ^5^ Department of Clinical Laboratory, Huadong Hospital, Shanghai Key Laboratory of Clinical Geriatric Medicine, Research Center on Aging and Medicine, Fudan University, Shanghai, 200040, China; ^6^ College of Pharmacy, Seoul National University, Seoul, 151–742, Republic of Korea

**Keywords:** neddylation, MLN4924 (pevonedistat), Cullin-RING ligases, prostate cancer, radiotherapy

## Abstract

Salvage radiotherapy (SRT) is the first-line treatment for prostate cancer patients with biochemical recurrence following radical prostatectomy, and new specific radiosensitizers are in urgent need to enhance SRT effect. MLN4924 (also known as Pevonedistat), a specific inhibitor of NEDD8-activating enzyme, has recently entered phase I/II clinical trials in several malignancies. By inhibiting cullin neddylation, MLN4924 inactivates Cullin-RING ligases (CRL), which have been validated as an attractive radiosensitizing target. In our study, we demonstrate that MLN4924 can be used as a potent radiosensitizer in hormone-resistant prostate cancer cells. We found that MLN4924 inhibited cullin neddylation and sensitized prostate cancer cells to irradiation (IR). Mechanistically, MLN4924 enhanced IR-induced G2 cell-cycle arrest, by inducing accumulation of WEE1/p21/p27, three well-known CRL substrates. Importantly, siRNA knockdown of WEE1/p21/p27 partially abrogated MLN4924-induced G2 cell-cycle arrest, indicating a causal role of WEE1/p21/p27 in MLN4924-induced radiosensitization. Further mechanistic studies revealed that induction of DNA damage and apoptosis also contributed to MLN4924 radiosensitization in hormone-resistant prostate cancer cells. Our findings lay the foundation for future application of MLN4924 as a potential radiosensitizer in hormone refractory prostate cancer (HRPC).

## INTRODUCTION

Prostate cancer is the most common form of cancer among males worldwide [1, 2]. Radical prostatectomy is one of the principal treatment modalities for localized prostate cancer [3]. Although surgery provides excellent control rates, about 20%–40% patients will experience biochemical recurrence within 5 years, presenting with increasing serum prostate-specific antigen (PSA) levels, without any evidence of clinical failure [4–9]. Within 5 years of prostatectomy, one in four men receive salvage radiotherapy (SRT), which entails external beam radiation to the prostate bed [10]. In patients exhibiting PSA recurrence, SRT is considered to be the only curative treatment. However, retrospective studies have showed that for patients treated with SRT, 5-year PSA recurrence-free survival (PRFS) rate is only 40%–50.1%, which is far from satisfaction [11–13]. Besides, with years of medical or surgical castration treatment, the relapse cells are always characterized by hormone-resistance. In order to improve survival benefit of SRT, efficient radiosensitizing agents against hormone-resistant prostate cancer cells are urgent to be identified.

Protein neddylation is a newly characterized protein posttranslational modification in eukaryotic cells by adding NEDD8, an ubiquitin-like molecule, to target proteins. The process is catalyzed by a three-step enzymatic cascade mediated by NEDD8-activating enzyme (NAE, E1), NEDD8-conjugating enzyme E2 (UBC12 or UBE2F) and substrate-specific E3 ligases [14–16]. Recently, MLN4924 (a specific inhibitor of NAE) has demonstrated its inhibitory activity against Cullin-RING E3 ligases (CRL) by blocking cullin neddylation. CRL, also known as SKP1-Cullin-F-box (SCF) for its founding member, is the largest multiunit E3 ubiquitin ligase family in cells [17]. Intensive studies have shown that the activation of CRL requires neddylation modification of its essential subunit cullin [18]. Thus, by inhibiting the neddylation pathway and blocking cullin neddylation, MLN4924 inactivates CRL, inducing diverse tumor-suppressive biological processes. Previous studies showed that inhibition of CRL by MLN4924 could act as a novel radiosensitizing agent in pancreatic, breast, and colorectal cancer cells [19–21]. Similarly, the genetic inactivation of CRL by knockdown of RBX1 or RBX2, two family members of the RING component of CRL, also induced tumor cell radiosensitization [22, 23]. These findings indicate that neddylation-CRL pathway serves as an attractive radiosensitizing target.

Based on these findings, we hypothesize that MLN4924 may sensitize hormone-resistant prostate cancer cells to radiation. As detailed below, we find that MLN4924 indeed acts as a radiosensitizing agent by enhancing G2 cell-cycle arrest, DNA damage and apoptosis by stimulating the accumulation of several CRL substrates. Our results lay the foundation for future development of MLN4924 as a radiosensitizing agent for hormone-resistant prostate cancer.

## RESULTS

### MLN4924 sensitized hormone-resistant prostate cancer cells to radiation

**Figure 1:**
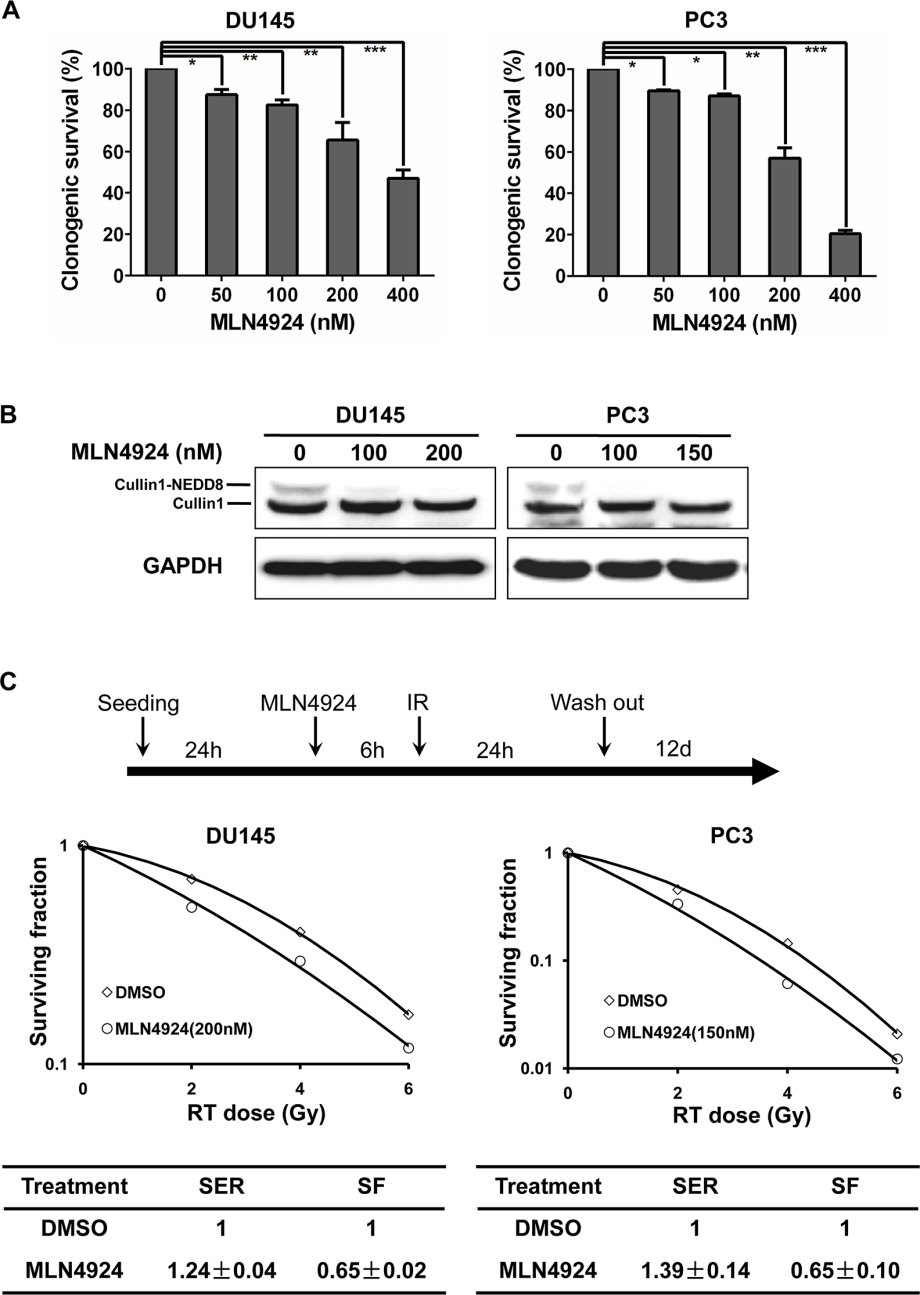
MLN4924 sensitized prostate cancer cells to radiation (**A**) MLN4924 inhibited clonogenic cell survival of prostate cancer cells. DU145 and PC3 were seeded in 60-mm dish in duplicate and treated with various concentrations of MLN4924. MLN4924 was washed out 24 hours post MLN4924-treatment and cells were cultured in MLN4924-free medium for additional 12 days. The colonies with more than 50 cells were counted, following crystal violet staining. (**B**) Deneddylation of cullin1 with the treatment of MLN4924. Subconfluent cells were treated with MLN4924 at indicated concentrations for 6 hours, followed by immunoblotting (IB) analysis using antibodies against cullin1, with GAPDH as a loading control. (**C**) Radiosensitization by MLN4924. Cells were seeded in 60-mm dish in duplicate and pretreated with MLN4924 at indicated doses (200 nM for DU145 and 150 nM for PC3) for 6 hours, followed by radiation at different doses up to 6 Gy. MLN4924 was washed out at 24 hours post radiation and cells were cultured in MLN4924-free medium for additional 12 days. Surviving fraction (SF) was calculated as the proportion of cells following irradiation to form colonies relative to that of untreated cells (mean ± SEM, *n* = 3). SER was calculated as the ratio of the mean inactivation dose under untreated control conditions divided by the mean inactivation dose after MLN4924 treatment. Shown is mean ± SEM, (*n* = 3): *P < 0.05, **P < 0.01, ***P < 0.0001. IR, irradiation.

To determine whether MLN4924 could enhance radiation efficacy, we performed radiosensitization experiments in two hormone-resistant prostate cancer cell lines, DU145 and PC3 [24, 25]. Firstly, we determined the sensitivity of the two prostate cancer cell lines to MLN4924 as a single agent. In a standard clonogenic survival assay, MLN4924 caused a dose-dependent inhibition of colony formation with an IC50 of 200–400 nM (Figure 1A). On the basis of IC50, we then measured a lower concentration of MLN4924 to abrogate neddylation of cullin, and found that MLN4924 at 200 nM/150 nM (for DU145/PC3 respectively) for 6 hours was efficient to block cullin neddylation completely (Figure 1B). We therefore used this dosing regimen in the following radiosensitization experiment. As shown in Figure 1C, MLN4924 caused a remarkable radiosensitizing effect in DU145 cells with a sensitivity enhancement ratio (SER) of 1.24, and in PC3 cells with a SER of 1.39. These results demonstrate that MLN4924 is an effective radiosensizer in hormone-resistant prostate cancer cells.

### MLN4924 significantly enhanced irradiation (IR)-induced G2 cell-cycle arrest, with accumulation of WEE1/p21/p27

**Figure 2:**
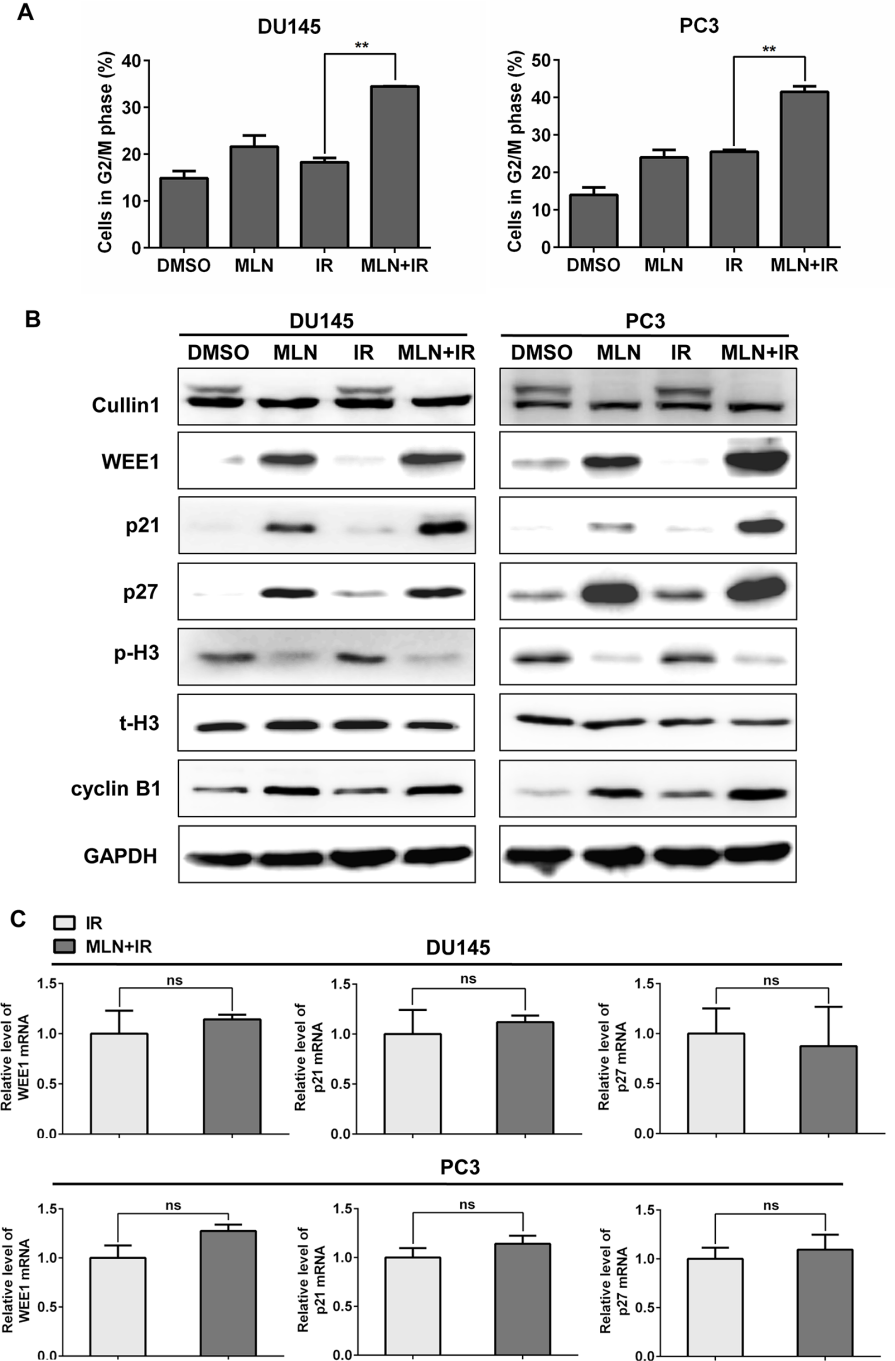
MLN4924 enhanced radiation-induced G2 arrest and accumulation of WEE1/p21/p27 in DU145 and PC3 cells (**A** and **B**) MLN4924-IR induced G2 arrest and accumulation of WEE1/p21/p27. Subconfluent cells were treated with MLN4924 (DU145 at 200 nM and PC3 at 150 nM) or IR (4 Gy) or MLN4924+IR, followed by cell cycle profile analysis (A), and IB analysis (B), using antibodies against cullin1, WEE1, p21, p27, p-H3, t-H3, and cyclin B1, with GAPDH as a loading control. (**C**) MLN4924-IR had little effect on the transactivation of WEE1/p21/p27. DU145 and PC3 cells were treated with IR (4 Gy) or MLN4924 (200 nM for DU145 and 150 nM for PC3) + IR (4Gy) for 6 hours, then subjected to real-time PCR for WEE1/p21/p27 with GAPDH as an internal control (*n* = 3). Shown is mean ± SEM, (*n* = 3): *P < 0.05, **P < 0.01, ***P < 0.0001. MLN, MLN4924; IR, irradiation.

To elucidate the underlying mechanisms for MLN4924 radiosensitization, cell-cycle profile was first determined using PI staining and FACS analysis. The two prostate cancer cell lines, DU145 and PC3, were treated with DMSO, MLN4924, IR, or MLN4924- IR, respectively. As shown in Figure 2A, MLN4924 remarkably enhanced the IR-induced G2 arrest in both DU145 (IR at 17% vs. MLN4924-IR at 34%) and PC3 (IR at 26% vs. MLN4924-IR at 43%) cells.

Previous studies have demonstrated a causal role of cell cycle inhibitors WEE1/p21/p27, three well-known substrates of CRL, in the induction of G2 cell-cycle arrest upon MLN4924 treatment [26]. To further investigate the mechanisms underlying G2 cell-cycle arrest, we measured the levels of these proteins in different treatment groups, and found that MLN4924-IR caused a further increase of WEE1/p21/p27 in both DU145 and PC3 cells compared to IR alone (Figure 2B). Meanwhile, the increased expression of WEE1, a well-defined inhibitor of G2-M phase transition [27], and decreased expression of p-H3, a hallmark of M phase cells [28], indicated that cells were arrest at G2 phase and failed to enter M phase [27]. These results suggest that WEE1/p21/p27 may contribute to MLN4924 radiosensitization in hormone-resistant prostate cancer cells.

Given that MLN4924-IR induced accumulation of WEE1/p21/p27, we tested whether MLN4924-IR regulated the expression of these proteins at transcriptional level. As shown in Figure 2C, MLN4924-IR had little effect on the transactivation of WEE1/p21/p27 compared to IR, determined by real-time PCR analyses for mRNA quantification. We therefore concluded that MLN4924-IR-induced accumulation of WEE1/p21/p27 was not regulated at mRNA level.

### MLN4924 inhibited the turnover of WEE1/p21/p27 proteins

**Figure 3:**
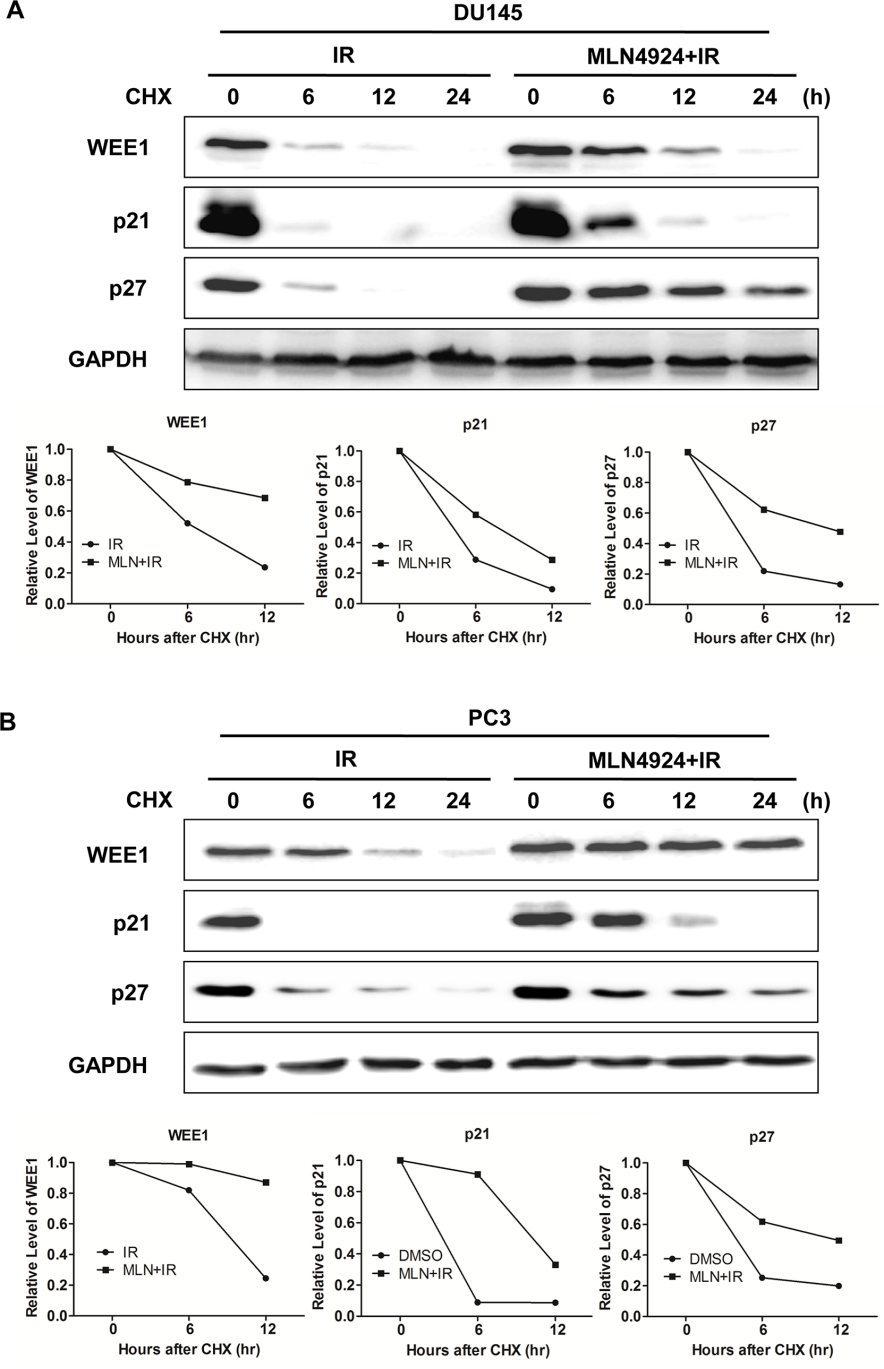
MLN4924-IR combination regulated degradation of WEE1/p21/p27 (**A** and **B**) Combination treatment extended the half-life of WEE1/p21/p27. DU145 and PC3 were pretreated by 1 μM MLN4924 for 12 hours, and then switched to new medium containing 25 μg/ml cycloheximide. Meanwhile, cells were treated with either IR (4 Gy), or MLN4924 (200 nM for DU145 and 150 nM for PC3) + IR for indicated time points, followed by IB using antibodies against cullin1, WEE1, p21, and p27, with GAPDH as a loading control.

To determine underlying mechanism of WEE1/p21/p27 regulated by MLN4924-IR, we further applied cycloheximide to block protein translation and determined WEE1/p21/p27 turnover upon MLN4924-IR compared to IR alone. As shown in Figure 3, MLN4924-IR significantly delayed WEE1/p21/p27 turnover and extended the half-life of WEE1/p21/p27 compared to IR alone in both DU145 and PC3 cells. These findings demonstrated that MLN4924-IR induced the accumulation of WEE1/p21/p27 by stabilizing those proteins.

### MLN4924-IR-induced G2 cell-cycle arrest was attributed to accumulation of WEE1/p21/p27

**Figure 4:**
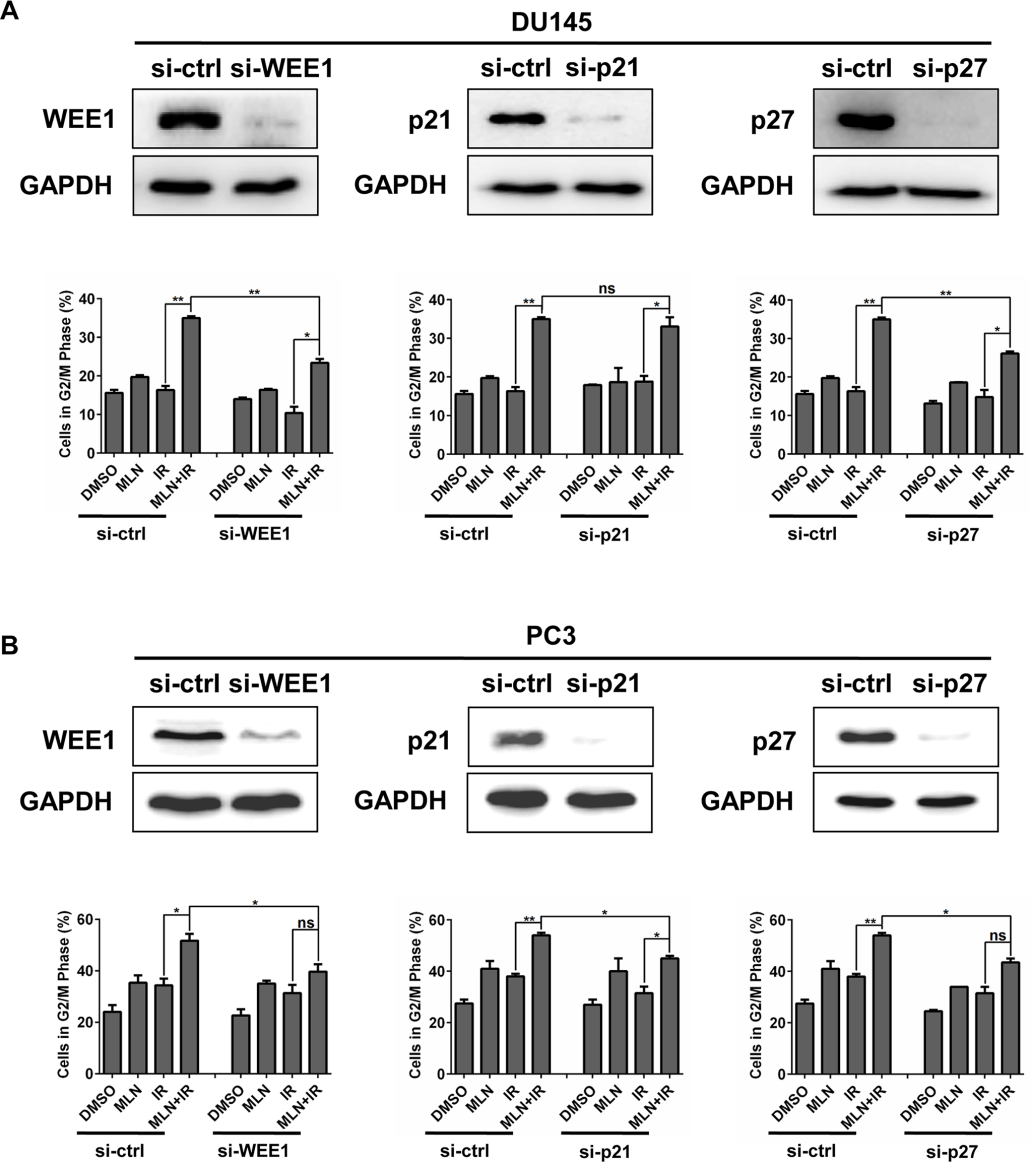
IR-enhanced G2 arrest was partially rescued by siRNA knockdown of WEE1/p21/p27 DU145 and PC3 cells were transfected with siRNA oligonucleotides targeting WEE1/p21/p27 respectively. Forty-eight hours later, one portion of cells was subjected to FACS analysis (**A** and **B**); the other portion was for IB analysis (A and B). Shown is mean ± SEM, (*n* = 3): *P < 0.05, **P < 0.01, ***P < 0.0001.

Considering that MLN4924 enhanced IR-induced G2 cell-cycle arrest and accumulation of WEE1/p21/p27, we determined whether accumulated WEE1/p21/p27 played a critical role in G2 cell-cycle arrest by down-regulating the expression of WEE1/p21/p27 via siRNA silencing in DU145 and PC3 cells. Cell-cycle profile analysis revealed that cells arrested at G2 phase were notably reduced upon WEE1 and p27 knockdown in both tested cell lines (Figure 4A and 4B). Similarly, p21 knockdown also reduced G2 population, significantly in PC3 cells and modestly in DU145 cells (Figure 4A and 4B). Thus, these results suggest a causal effect of WEE1/p21/p27 accumulation on MLN4924-IR-induced G2 cell-cycle arrest in prostate cancer cells.

### MLN4924 enhanced IR-induced apoptosis and DNA damage

**Figure 5:**
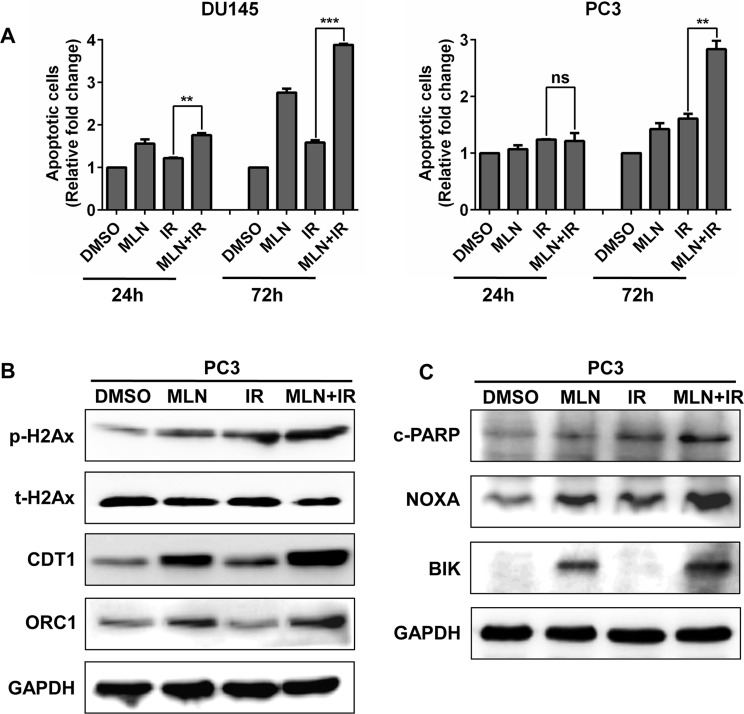
MLN4924 enhanced IR-induced apoptosis and DNA damage (**A** and **C**) MLN4924-IR induced more apoptotic cells than IR alone. Subconfluent cells were treated with MLN4924 (DU145 at 200 nM and PC3 at 150 nM) or IR (4 Gy) or MLN4924+IR, 72 hours later, one portion of cells was subjected to Annexin V-FITC/PI double-staining analysis for apoptosis (A); the other portion was for IB analysis (C). (B) MLN4924 enhanced DNA damage in IR-treated cells. DU145 and PC3 cells were treated with MLN4924 (DU145 at 200 nM and PC3 at 150 nM) or IR (4 Gy) or MLN4924+IR for 24 hours, followed by IB analysis, using antibodies against p-H2Ax, t-H2Ax, CDT1, and ORC1, with GAPDH as a loading control. Shown is mean ± SEM, (*n* = 3): *P < 0.05, **P < 0.01, ***P < 0.0001. MLN, MLN4924; IR, irradiation.

To further investigate the outcome of the cells arrested at G2 phase, we detected apoptotic cells in each treatment group. As shown in Figure 5A, MLN4924 increased the IR-induced apoptotic population at later time points (72 hr) in both tested cell lines. Previous studies have demonstrated that MLN4924 can enhance IR-induced DNA damage [19], we therefore determined DNA double-strand breaks (DSBs) by measuring the levels of p-H2AX protein in PC3 cells. As shown in Figure 5B, compared to treatment alone, a higher level of p-H2AX was detected in MLN4924-IR treatment group. Mechanistically, DNA licensing proteins CDT1 and ORC1, two classical substrates of CRL whose accumulation induces DNA replication stress and DNA damage [29], were significantly increased in MLN4924-IR (Figure 5B). Furthermore, we found that pro-apoptotic proteins NOXA and BIK were notably accumulated upon MLN4924-IR treatment when compared to MLN4924 or IR treatment alone (Figure 5C). These findings indicated that the induction of DNA damage and apoptosis plays an important role in MLN4924-induced radiosensitization.

### Genetic inactivation of neddylation-CRL pathway sensitized hormone-resistant prostate cancer cells to radiation

**Figure 6:**
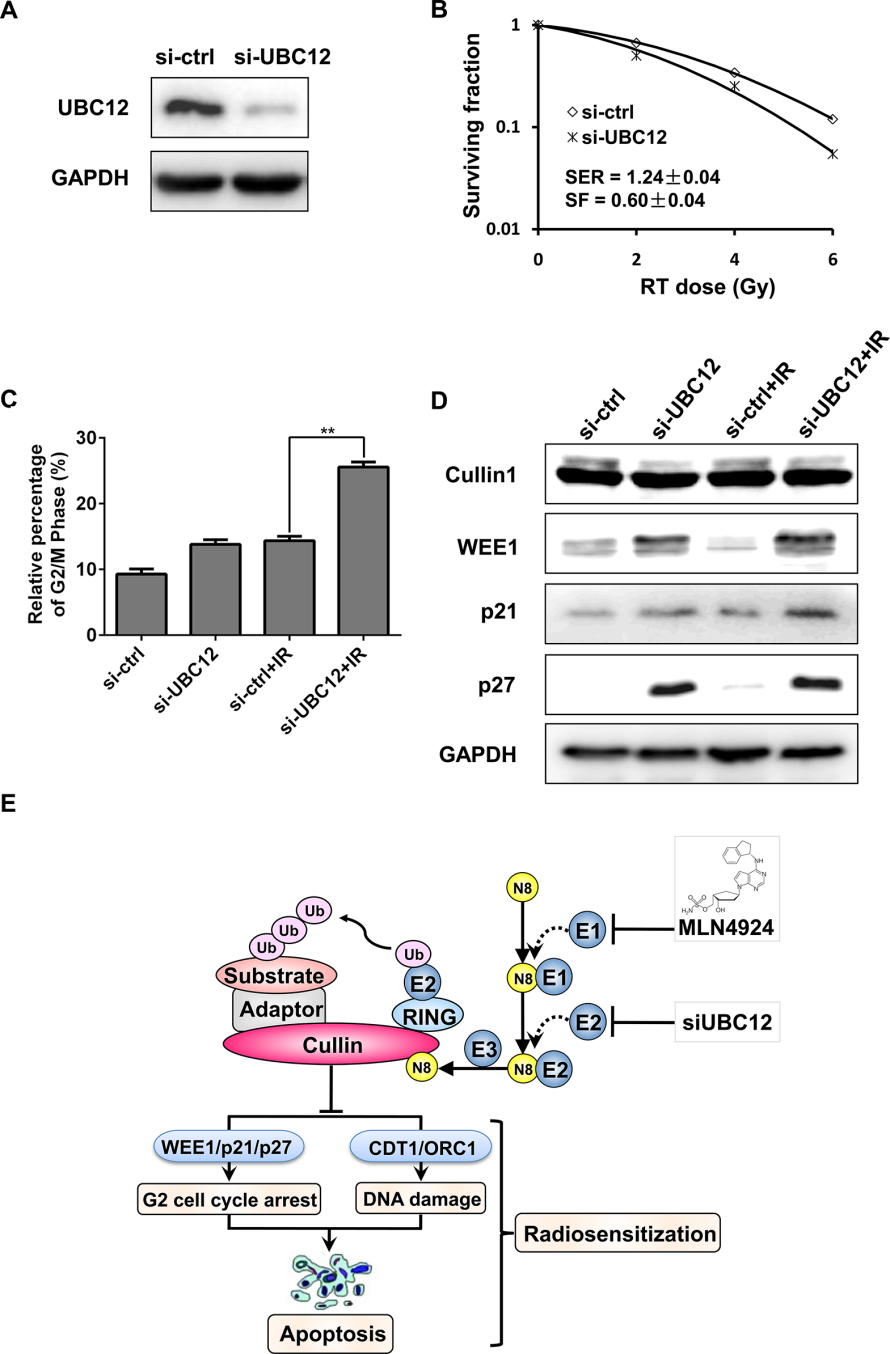
UBC12 silencing sensitized prostate cancer cells to radiation (**A**–**D**) si-UBC12 sensitized DU145 to radiation. DU145 were transfected with siRNA oligonucleotides targeting UBC12. Forty-eight hours later, one portion of cells was plated for clonogenic assay (B), one portion was for FACS analysis (C), and still other portion was subjected for IB analysis (A and D). Shown is mean ± SD (*n* = 3). (**E**) Radiosensitization by inactivation of CRL. Inactivation of CRL by specific small-molecule MLN4924, as well as si-UBC12, which inhibited cullin neddylation, lead to accumulation of CRL substrates. Accumulated substrates, including WEE1/p21/p27, CDT1/ORC1 and NOXA/BIK that trigger G2 cell-cycle arrest, DNA damage and apoptosis, were related to MLN4924 mediated radiosensitization in prostate cancer cells. Shown is mean ± SEM, (*n* = 3): *P < 0.05, **P < 0.01, ***P < 0.0001. MLN, MLN4924; IR, irradiation; N8, NEDD8.

The above results indicated that by targeting NEDD8-activating enzyme (E1, NAE) pharmacologically, MLN4924 effectively sensitized hormone-resistant prostate cancer cells to radiation. To further verify radiosensitization by inhibiting neddylation pathway, the expression of NEDD8-conjugating enzyme E2 (UBC12) was downregulated by siRNA konckdown. As expected, UBC12 silencing significantly sensitized DU145 to radiation with a SER of 1.24 (Figure 6A and 6B). Mechanistic studies revealed that knockdown of UBC12 remarkably enhanced the IR-induced G2 cell-cycle arrest (Figure 6C), and similarly, siUBC12-IR combination caused an increased expression of WEE1/p21/p27 in DU145 cells (Figure 6D). Taken together, targeting neddylation pathway by pharmacologic or genetic methods can effectively sensitize hormone-resistant prostate cancer cells to radiation.

## DISCUSSION

Salvage radiotherapy (SRT) is recognized as the sole approach affording an opportunity of a cure to patients with localized prostate cancer who develop PSA recurrence after radical prostatectomy and years of castration treatment [13]. However, the long-term PRFS for patients who undergo SRT is still far from satisfactory [30]. Thus, development of radiosensitizing agents against hormone-resistant prostate cancer cells is in high demanding for the improvement of SRT outcome.

The major finding of this study is that targeting neddylation-CRL axis pharmacologically (MLN4924) or genetically (siUBC12) can sensitize hormone-resistant prostate cancer cells to radiation. Mechanistically, inhibition of neddylation pathway enhanced IR-induced G2 cell-cycle arrest, which was attributed to accumulated WEE1/p21/p27 (Figure 6E), as evidenced by (a) MLN4924 (or siUBC12)-IR further enhanced IR-induced G2 cell-cycle arrest (Figures 2A and 6C); (b) MLN4924 (or siUBC12)-IR further enhanced the accumulation of WEE1/p21/p27 by stabilizing these cell cycle inhibitors as CRL substrates (Figures 2B and 6D); and (c) more importantly, knockdown of WEE1/p21/p27 individually attenuated G2 cell-cycle arrest (Figure 4), indicating that the accumulation of those CRL substrates contributed to cell cycle arrest synergistically. Moreover, MLN4924-IR-enhanced DNA damage and apoptosis may also contribute to MLN4924-induced raidosensitization.

Previous studies have reported that inactivation of neddylation-CRL pathway by MLN4924 sensitizes other types of human cancer cells to radiation [23]. In pancreatic cancer cells, MLN4924 caused accumulation of CDT1, WEE1, and NOXA, in parallel with an enhancement of radiation-induced DNA damage, aneuploidy, G2 arrest and apoptosis [19]. While in breast cancer cells, the radiosensitization of MLN4924 appeared to mainly depend on the accumulation of p21, associating with cell growth inhibition and apoptosis [20]. In our study, MLN4924 caused accumulation of WEE1/p21/p27, CDT1/ORC1 and NOXA/BIK, which were associated with MLN4924-IR-enhanced G2 cell-cycle arrest, DNA damage and apoptosis in prostate cancer cells. Collectively, MLN4924 displays remarkable radiosensitizating activity in human malignancies due to the inactivation of CRL and accumulation of a subset of tumor-suppressive CRL substrates.

In summary, our study demonstrates that inactivation of neddylation-CRL pathway serves as a potent radiosensitizing strategy in prostate cancer cells by triggering G2 cell-cycle arrest, DNA damage and apoptosis. These findings lay the foundation for future application of neddylation-CRL inhibitors (e.g. MLN4924) as a novel radiosensitizer in hormone-resistant prostate cancer.

## MATERIALS AND METHODS

### Cell culture and reagents

Two human DU145 and PC3 prostate cancer cell lines were purchased from American Type Culture Collection and grown in RPMI1640 with 10% fetal bovine serum. Neddylation pathway inhibitor MLN4924 and protein biosynthesis inhibitor cycloheximide (CHX) were each dissolved in dimethyl sulfoxide (DMSO) and kept in −20°C before use.

### Radiation exposure and clonogenic assay

Cells were seeded in 60-mm dishes at proper cell densities in duplicate and exposed to different doses of radiation (SARRP, Gulmay Medical) after 6 hours pre-treatment with MLN4924. MLN4924 was washed away afterwards, followed by incubation at 37°C for 12 days. Survival curves were fitted using the linear-quadratic equation, and the mean inactivation dose was calculated [31].

### Immunoblotting (IB)

Cells were harvested and cell lysates were extracted for immunoblotting as described [32], using antibodies against cullin1, p21, p27, WEE1, phospho-histone H3 (p-H3), total-histone H3 (t-H3), cyclin B1, phospho-H2AX (p-H2Ax), total-H2AX (t-H2Ax), CDT1, ORC1, cleaved PARP (c-PARP), NOXA, BIK, UBC12, and GAPDH.

### RNA interference

The siRNA oligonucleotides are as follows: si-WEE1 (5′–GAGGCUGGAUGGAUGCAUUUU–3′) [19], si-p21 (5′–GUGGACAGCGAGCAGCUGAUU–3′) [20], si-p27 (5′–CCGACGAUUCUUCUACUCA–3′) [33], si-UBC12 (5′–GGGCUUCUACAAGAGUGGGAAGUUU–3′) [34] and si-Control (5′–UUCUCCGAACGUGUCACGUUU– 3′) [35]. The oligoes were purchased from GenePharma (Shanghai, China). Cells were transfected with siRNA using Lipfectamine 2000 according to the manufacturer's instructions and split 48 hours later. One portion was used for clonogenic assay, and the other portion for immunoblotting (IB) or fluorescence-activated cell sorting profile.

### Propidium iodide (PI) staining and fluorescence-activated cell sorting (FACS) analysis

Cells were treated with MLN4924, or exposed to IR or in combination. After fixed in ice-cold 70% ethanol at −20°C overnight, cells were strained with propidium iodide (PI; 36 μg/mL; Sigma) at 37°C for 15 minutes, and then analyzed cell-cycle profile by CyAn ADP (Beckman Coulter). Data were analyzed with ModFit LT software [32].

### Detection of apoptosis

Cells were treated with the indicated concentration of MLN4924 for 24 and 72 hours. Apoptosis was determined with the Annexin V-FITC/PI Apoptosis Kit (BioVision, Inc. Milpitas, California) according to manufacturer's instructions.

### Real-time polymerase chain reaction (real-time PCR) analyses

Total RNA was isolated using the Trizol reagent (Invitrogen, Carlsbad, CA) according to the manufacturer's instructions and treated with RNase-free DNase. The reverse transcription reaction was performed on 1 μg of total RNA per sample using the PrimerScript reverse transcription reagent kit (TaKaRa, Shiga, Japan) according to the manufacturer's protocol. After reverse transcription, the real-time polymerase chain reaction (PCR) was performed using the Power SYBR Green PCR MasterMix (Applied Biosystems, Foster City, CA) on the ABI 7500 thermocycler (Applied Biosystems) following the instrument manual. The sequences of the primers are as follows: Human β-actin: forward 5′-TGACGTGGACATCCGCAAAG-3′, reverse 5′-CTGGAAGGTGGACAGCGAGG-3′ [35] ; Human WEE1: forward 5′-ATTTCTCTGCGTGGGCAGAAG-3′, reverse 5′-CAAAAGGAGATCCTTCAACTCTGC-3′ [36]; Human p21: forward 5′-GACTCTCAGGGTCGAAAA CG-3′, reverse 5′-GGATTAGGGCTTCCTCTTGG-3′ [37]; Human p27: forward 5′-TCCGGCTAACTCTGAGGAC AC-3′, reverse 5′-TGTTTTGAGTAGAAGAATCGTC GGT-3′ [37].

### Statistical analysis

ANOVA were used with SPSS (Statistical Product and Service Solution) software for statistical comparisons involving multiple groups, followed by SNK post hoc test to determine significance of each two group (*p* < 0.05). The unpaired 2 - tailed *t* test was performed for the comparison of two groups, and the level of significance was set at ***P < 0.05, ***P < 0.01, ***P < 0.0001, using GraphPad Prism5 software.
